# The quality of Cochrane systematic reviews of acupuncture: an overview

**DOI:** 10.1186/s12906-020-03099-9

**Published:** 2020-10-14

**Authors:** Zhaochen Ji, Junhua Zhang, Francesca Menniti-Ippolito, Marco Massari, Alice Josephine Fauci, Na Li, Fengwen Yang, Mingyan Zhang

**Affiliations:** 1 Evidence-Based Medicine Center, Tianjin University of Traditional Chinese Medicine, Tianjin, China; 2 Italian National Institute of Health, Rome, Italy; 3 School of Social and Political Sciences, Institute of health and wellbeing, University of Glasgow, Glasgow, UK

**Keywords:** Cochrane systematic reviews of acupuncture, Methodological quality, Overview, AMSTAR 2

## Abstract

**Background:**

Many systematic reviews of clinical trials on acupuncture were performed within the Cochrane Collaboration, the evidence-based medicine (EBM) most recognized organization. Objective of the article was to systematically collect and identify systematic reviews of acupuncture published in the Cochrane Library and assess their quality from a methodological perspective.

**Methods:**

A comprehensive literature search was performed in the Cochrane Database of Systematic Reviews to identify the reviews of acupuncture conducted until June 2019. The methodological quality of the included reviews was assessed using the AMSTAR 2 checklist, an evaluation tool for systematic reviews.

**Results:**

Out of a total of 126 eligible reviews, 50 systematic reviews were included. According to the AMSTAR 2, 52% of Cochrane Systematic Reviews (CSRs) were of low quality, due to the presence of one or more weaknesses in at least one of the domains defined as critical for the methodological quality assessment. The less satisfied critical domain was inadequate investigation and discussion of publication bias. Declaration of potential sources of conflict of interest, and funding of the authors of the review and of the included studies were other important weaknesses.

**Conclusions:**

The main methodological flaws in the included CSRs were related to topics of relatively new concern in the conduction of systematic reviews of the literature**.** However, both, lack of attention about retrieval of negative studies, and statements about conflict of interests are crucial point for the evaluation of therapeutic interventions according to EBM methodology.

## Background

Acupuncture represents, together with herbal medicine, the main treatment used in Traditional Chinese Medicine (TCM). TCM has been practiced for thousands of years and is widely used in Western countries. Nevertheless, there is still debate on the efficacy of acupuncture according to the evidence-based medicine (EBM) paradigm. In the literature, different, sometimes conflicting, results have been published for different conditions [1–4]. Different results might come from different study designs, different interventions, and different settings.

Systematic reviews can provide objective and reliable evidence, which is useful for clinical practice. Cochrane Systematic Reviews (CSRs) are regarded as the highest level of evidence; however, although they represent the best quality systematic reviews, it cannot be excluded that CSRs on acupuncture could present some methodological flaws [5]. Since 1998, the Cochrane Database of Systematic Reviews (CDSR) has included many systematic reviews of acupuncture. The aim of this study was to systematically collect systematic reviews of acupuncture published in the Cochrane Library and to assess their methodological quality.

## Methods

All eligible acupuncture-related systematic reviews in CDSR, up to June 2019, were searched. The searching query was “Acupuncture [Mesh] or Acupuncture [title, abstract, keywords]”.

All types of acupuncture were included, such as “acupuncture”, “auricular acupuncture”, “electro acupuncture”, “scalp acupuncture”, “manual acupuncture (needle acupuncture)”, “warming needle (heat needle)”, “laser acupuncture”, “acupressure” and “injection acupuncture”. All outcome measures were eligible for inclusion in this review. Protocols of CSRs and withdrawn systematic reviews were excluded.

The extracted data included: title, author, nationality, year of publication, author’s affiliation, type of disease, type of literature included, interventions, adverse reactions, outcome measures, literature searching database, update, literature quality assessment of clinical trials and study result (positive / negative).

Two reviewers (Zhaochen Ji and Na Li) extracted the data and cross-checked the consistency; disagreements were resolved by discussion with a third researcher (Junhua Zhang).

The methodological quality of CSRs was assessed using the AMSTAR 2 tool (A MeaSurement Tool to Assess Systematic Reviews) [6]. The AMSTAR 2 checklist requires reviewers to answer: 1) yes, 2) no, 3) partial yes or 4) no meta-analysis conducted, on 16 items or domains. Seven of the 16 items are by default labeled as “Critical domains” because they can critically affect the validity of a review and its conclusions, the “Critical domains” 2, 4, 7, 9, 11, 13, and 15 are shown in Table 1. As recommended by AMSTAR 2 authors, we rated the overall quality of each included review on the basis of four levels: *High* (No or one non-critical weakness: the systematic review provides an accurate and comprehensive summary of the results of the available studies that address the question of interest), *Moderate* (More than one non-critical weakness: the systematic review has more than one weakness but no critical flaws and it may provide an accurate summary of the results of the available studies that were included in the review), *Low* (One critical flaw with or without non-critical weaknesses: the review has a critical flaw and may not provide an accurate and comprehensive summary of the available studies that address the question of interest), *Critically low* (More than one critical flaw with or without non-critical weaknesses: the review has more than one critical flaw and should not be relied on to provide an accurate and comprehensive summary of the available studies) [6].

**Table 1:** Critical domains of AMSTAR 2

Item	Critical Domains
2	Did the report of the review contain an explicit statement that the review methods were established prior to the conduct of the review and did the report justify any significant deviations from the protocol?
4	Did the review authors use a comprehensive literature search strategy?
7	Did the review authors provide a list of excluded studies and justify the exclusions?
9	Did the review authors use a satisfactory technique for assessing the risk of bias (RoB) in individual studies that were included in the review?
11	If meta-analysis was performed did the review authors use appropriate methods for statistical combination of results?
13	Did the review authors account for RoB in individual studies when interpreting/discussing the results of the review?
15	If they performed quantitative synthesis did the review authors carry out an adequate investigation of publication bias (small study bias) and discuss its likely impact on the results of the review?

## Results

### Literature search

**Fig. 1:**
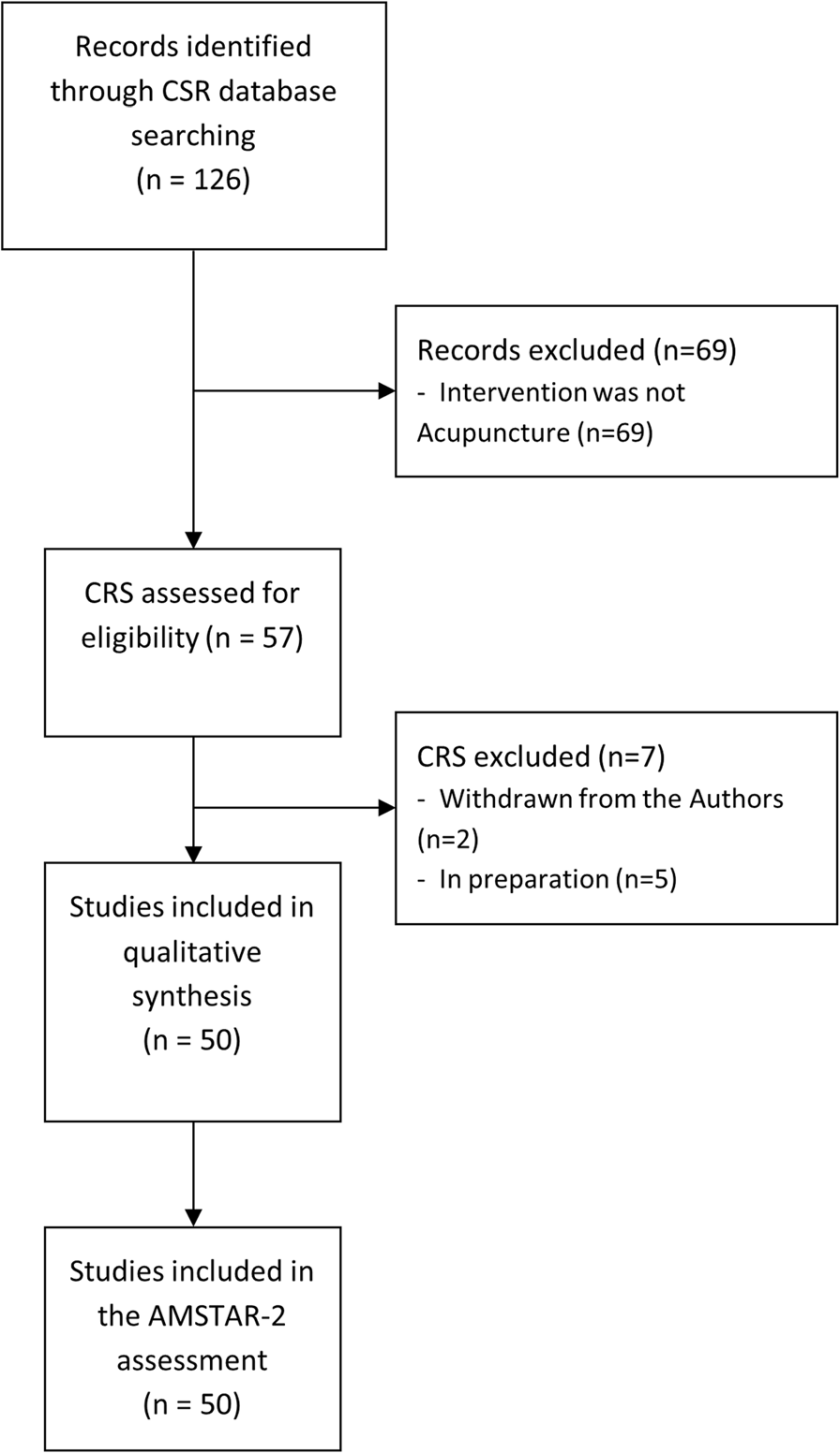
Flowchart of CSR selection

Using the described searching strategy, we obtained 126 potentially relevant reviews. Sixty-nine reviews were excluded because the intervention was not “acupuncture”. Fifty-seven reviews were identified; of these, 2 reviews were withdrawn by the authors because “new citations are required and conclusions have changed” and because “the authors of this review have been unable to complete the update within the recommended two-year period”; 5 reviews from China were in preparation and only the protocol were present in the database. Finally, 50 CSRs were included (Fig. 1).

The characteristics of the included CSRs are described in Table S1 [7–56].

All types of studies included in the CSRs were RCTs. In the 50 concluded reviews, a total of 837 RCTs with 92,903 participants (range 33–7667) were included.

The included CSRs were published from 2002 to June 2019, at an average of 3 published CSRs each year, with a peak of 8 CSRs published in 2018. Regarding the geographical distribution of the first author: 19 (38%) were from China, 13 (26%) were from Australia, 5 (10%) were from UK, and the remaining authors were from United States, Canada, South Korea, and Germany.

With reference to the year of publication in the Cochrane database, only 13 reviews were updated within 5 years after publication [8–11, 13, 31, 36–38, 43, 49, 51, 54].

The number of databases used for literature search in the included CSRs varied from 3 to 28 (mean: 9). All the included systematic reviews searched databases in English, while 31 reviews (62%) also searched Chinese databases. Among the English databases, EMBASE was the most searched (48 reviews), while MEDLINE was searched in 47 reviews. The Chinese databases searched were China National Knowledge Infrastructure [CNKI] (in 18 reviews), Vip Chinese science and technology journal database [VIP] (in 16 reviews), and SinoMed (in 15 reviews). The search frequencies of all the English and Chinese databases are shown in Table 2.

**Table 2:** English and Chinese databases searched

English Databases (***N*** = 50)	n (%)	Chinese Databases (N = 50)	n (%)
EMBASE	48 (96.0%)	CNKI	18 (36.0%)
MEDLINE	47 (94.0%)	VIP	16 (32.0%)
CENTRAL	37 (74.0%)	CBM (Chinese bio-medical database)	15 (30.0%)
CINAHL	31 (62.0%)	Wanfang	9 (18.0%)
AMED	28 (56.0%)	TCMLARS	4 (8.0%)
PsycInfo	14 (28.0%)	CJFD	3 (6.0%)
ICTRP	14 (28.0%)	CMCC	4 (8.0%)
Clinicaltrials.gov	10 (20.0%)	SinoMed	3 (6.0%)
Cochrane Library	7 (14.0%)	Chinese Acupuncture Trials Register	3 (6.0%)
PubMed	6 (12.0%)	China Master Theses Full-text Database	2 (4.0%)
NCCAM	5 (10.0%)	China Proceedings of Conference Database	2 (4.0%)
LILACS	3 (6.0%)	Index to Taiwan Periodical Literature System	2 (4.0%)
mRCT	4 (8.0%)	China Doctor Dissertation Full-text Database	2 (4.0%)
Korean Medical Database	4 (8.0%)	Chinese Clinical Trials Registry	2 (4.0%)
Others ^a^	< 4 (< 8.0%)	China dissertation database	2 (4.0%)
		Index to Chinese Periodical Literature	2 (4.0%)
		Others ^b^	< 2 (< 4.0%)

^a^ Cochrane Menstrual Disorders and Subfertility Group Trials Register, Current Controlled Trials, Dissertation Abstracts International: 3 (7.5%); Cochrane Controlled Trials Register, Database of Abstracts of Reviews of Effectiveness (DARE), Korean Medical Database, National Research Register, NIH-Clinical Trials Database, OVID, SCISEARCH, SIGLE, SPORTDiscus: 2 (5%); BIOSIS Previews, A database search of controlled clinical trials published in Japan, ACP Journal Club, ACUBRIEFS, CISCOM, Cochrane Bone Joint and Muscle Trauma Group Specialised Register, Cochrane Complementary Medicine Field Specialized Register, Cochrane Complementary Medicine Field’s Trials Register, Cochrane Epilepsy Group Specialised Register, Cochrane Incontinence Group Specialised Register, Cochrane Inflammatory Bowel Disease and Functional Bowel Disorders Review Group Specialized Register, Cochrane Injuries Group Specialised Register, Cochrane Stroke Group Trials Register, Cumulative Index to Nursing and Allied Health, EBSCOhost, HealthSTAR, HSRProj via the National Library of Medicine, ISI Proceedings, ISI Web of Science, Japana Centra Revuo Medicina, J-East, MEDLINE in Process, MIDIRS, ProQuest Digital Dissertations, Science Citation Index, Stroke Trials Directory, ZETOC: 1 (2.5%)

^b^ Chinese Evidence-Based Medicine Database, CACP, China’s important Conference Papers Database, China dissertation database, Chinese Stroke Trials Register, Chinese Biomedical Retrieval System, CSTDB, Chinese Social Science Citation Index, Chinese Science and Technique Journals Database, China National Infrastructure, Index to Chinese Periodical Literature, TCMDS

The quality of the RCTs in the included CSRs was evaluated using different tools: 39 reviews referred to the bias risk assessment tool recommended by the Cochrane Handbook for Systematic Reviews, 2 reviews referred to the Jadad scale [17, 37], and one review referred to both [50]. In 8 reviews, the methodological quality assessment tools were not explicitly reported [8, 18, 20–22, 28, 54, 55].

### Treated conditions

The included CSRs focused on 50 different diseases or conditions. Eight reviews (16%) evaluated acupuncture for joint and muscle pain [8, 18, 20, 22, 23, 25, 34, 36], 5 reviews (9%) for obstetrics [11, 16, 41, 42, 44], 5 reviews (9%) for gynecological diseases [7, 31, 32, 43, 56], 3 reviews (4%) for stroke [51, 52, 54] and 2 reviews (4%) for smoking cessation [21, 48] The other diseases are listed in Table S1.

### Acupuncture techniques

Eighteen (35%) CSRs did not define and describe the acupuncture technique used, while in the remaining CSRs, several types of acupuncture were employed. The 19 different acupuncture techniques used were summarized in four categories (Table 3).

**Table 3:** The types and frequency of acupuncture interventions

Acupuncture Intervention	n	(%)
Traditional acupuncture with/without additional stimulation		
Traditional acupuncture	45	(88.2)
Electric acupuncture	17	(33.3)
Warm acupuncture	4	(7.8)
Non-acupoint acupuncture	3	(7.5)
Pyonex	1	(2)
Other acupoint stimulation		
Scalp needles	8	(15.7)
Dermal needle	2	(3.9)
Face acupuncture	1	(2)
Tongue acupuncture	1	(2)
Ear acupuncture	14	(27.5)
Non-penetrated acupoint stimulation		
Acupressure	16	(31.4)
Surface electrical stimulation	3	(5.9)
Laser needle	16	(31.4)
Magnetic needle	3	(5.9)
Moxibustion	9	(17.6)
Other stimulations		
Acupoint injection	3	(5.9)
Embedding	2	(3.9)
Acupoint bloodletting	1	(2)
Intradermal needling	1	(2)

The first category included traditional acupuncture, which consists of insertion of acupuncture needles in specific body points according to the traditional meridian theory. In clinical practice, small electrical charge and moxibustion are commonly added to traditional acupuncture (electroacupuncture and warm acupuncture). The second category of acupuncture included stimulation of defined body regions and points different from the traditional meridian theory, such as: scalp, dermal, face, tongue and ear. The third category was related to non-penetrated acupoint stimulation (meaning that no needle penetrates into the skin), such as acupressure, surface electrical stimulation, laser needle, magnetic needle, and moxibustion. The fourth category included other stimulations, such as acupoint bloodletting, intradermal needling, acupoint injection and embedding acupuncture. The interventions and controls are shown in Table S2.

### Outcomes and safety

In terms of the reported outcomes, different acupuncture types used for different conditions produced different results. The majority of outcomes were related to improvement in quality of life (60%) and pain relief (32%).

The conclusions of included CSRs can be summarized into four categories: 1) thirty-two reviews (64%) concluded that the evidence was not sufficient or the level of evidence was not adequate; 2) seven reviews (14%) reported positive results and found acupuncture to be effective in the treatment of chronic kidney disease [26], induction of labor [42], episodic migraine [32], tension-type headache [33], fibromyalgia [18], pain in childbirth [41], and nausea and vomiting after surgery [30]; 3) ten reviews (20%) reported “potential efficacy” for acute management and rehabilitation of traumatic brain injury [49], endometrial pain [56], osteoarthritis [34], schizophrenia [39], post stroke rehabilitation [52] breech presentation [16], side effects of chemotherapy or radiotherapy in persons with cancer [55], acute stroke [51], induction of labour [44], premenstrual syndrome [7]; and 4) one review reported negative results for irritable bowel syndrome [35].

With regard to acupuncture safety, 24 reviews (48%) reported adverse events in the included RCTs, eleven reviews (22%) did not report information about adverse events [8, 20, 22, 32–34, 37, 41, 42, 48, 50], and 15 reviews (30%) reported no adverse events [7, 9, 11, 17, 18, 21, 26, 28, 29, 38–40, 43, 49, 56]. Regarding the main adverse events, 9 reviews reported unbearable pain or pain caused by anxiety [12, 13, 15, 16, 36, 47, 51, 52, 54]; 5 reviews reported headache, dizziness or even syncope [14, 16, 23, 35, 51]; 2 reviews reported local infection [10, 23]; 3 reviews reported local fever or swelling [10, 31, 47]; 2 reviews reported blood stasis [19, 31]; one review reported allergy [30]; and one review reported ear irritation [47].

### Quality assessment

The evaluation of CSRs using the AMSTAR 2 scale is presented in Table S3.

Almost half of CSRs were of high or medium quality: 8 CSRs (16%) were assessed as high quality, and 16 CSRs (32%) were assessed as medium quality. The highest quality CSRs were related to neuropathic pain in adults [24], glaucoma [29], postoperative nausea and vomiting [30], acute hordeolum [10], premenstrual syndrome [7], carpal tunnel syndrome [15], symptomatic gastroparesis [38] and depression [45].

**Fig. 2:**
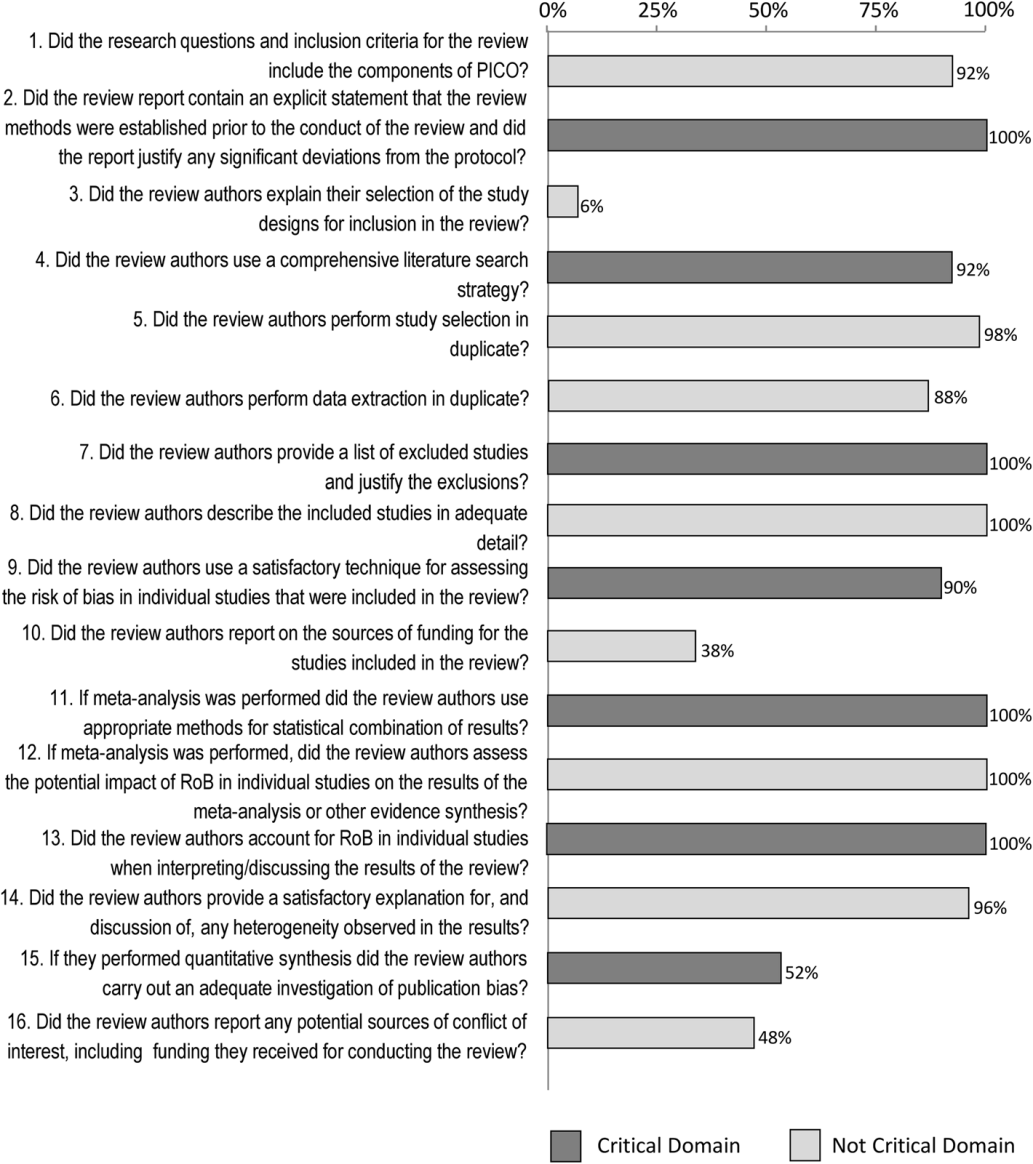
Percentage of AMSTAR scores by item (N = 50)

Analyzing Table S3 by column (Fig. 2), we could discriminate met and unmet items, which highlighted some critical methodological issues.

Six of the 16 AMSTAR 2 items were met by all the CSRs included in our study: critical domain 2 (pre-specification of the review protocol); critical domain 7 (justification for study exclusion); domain 8 (description of the included studies); critical domain 11 (appropriateness of meta-analytical methods); domain 12 (assessing of the potential impact of risk of bias); critical domain 13 (consideration of the risk of bias when interpreting the results of the review). In contrast, four items of the AMSTAR 2 were less commonly met, thus indicating some methodological weaknesses: 1) for domain 3 (explanation for the study design inclusion) (6.0%); 2) for domain 10 (reporting the sources of funding) (38.0%); 3) for critical domain 15 (investigation and discussion of publication bias) (52%); 4) for domain 16 (reporting of potential sources of conflict of interest) (48%).

## Discussion

For this overview, we identified and analyzed 50 acupuncture-related CSRs published up to June 2019. Many of the included systematic reviews were updated, starting from 1998 [32, 37]. The methodological quality of the systematic reviews included in the Cochrane library, assessed using the AMSTAR 2 tool, was medium to high in 48% of the included CSRs, according to the rating overall confidence in the results of the reviews. More than half of the reviews were of low quality, due to the presence of one or more weaknesses in at least one critical domain. The less satisfied critical domain of the AMSTAR 2 evaluation tool was the adequate investigation of publication bias (critical domain 15). However, regarding the assessment of publication bias a negative evaluation can be attributed to the year of publication (before 2007 assessment of publication bias was not common) and the number of trials included in the review: a minimum of 10 trials are usually required to perform a funnel plot, a graphical tool used to describe the presence of publication bias. In general, publication bias is a well-known issue in the Chinese literature [57–59].

Only 2 reviews resulted of critically low quality, both conducted and published in the early 2000s (2003 and 2005), with the following unmet critical domains of AMSTAR 2: comprehensive literature search; risk of bias, in particular publication bias.

However, the AMSTAR 2 evaluation pointed out some particular issues associated with the CSRs on acupuncture. In 8% of the reviews, authors did not use a comprehensive literature search strategy. Although most of the acupuncture RCTs were published in China, Chinese literature databases were not always searched, likely due to language limitations and accessibility of Chinese databases.

With regard to non-critical domain of the AMSTAR 2, the less met domains were number 10 (38%) and number 16 (48%), related respectively to funding and conflict of interest. In many of the studies included in the reviews, funding sources were not declared, this issue was also not often reported, so as the potential conflict of interest of authors that conducted the reviews.

In order to investigate if there was an improvement in the quality of newer reviews with regard to older ones, two sensitivity analyses were performed evaluating reviews published after 2010 and after 2015. In the first analysis 41 reviews were selected and the percentage of medium to high quality was increased to 56%, with a further increase to 65% in the last 5-year period. However, publication bias still remains the main issue to deal with. Five reviews did not adequately investigate or discuss the risk of publication bias, even if the number of included clinical trials were over the minimum number of 10 studies usually required.

Lack of retrieval of negative studies in the literature is a crucial point for the evaluation of therapeutic interventions according to EBM methodology; thus, more efforts should be put in the retrieval of grey literature and negative results. To advance in science a cultural change is needed, negative results are as important as positive ones to improve knowledge on efficacy of treatments.

Due to the large variety of intervention/control measures and outcomes in each CSR, it is difficult to reach general conclusions about clinical efficacy of acupuncture. However, according to the results of some CSRs, acupuncture seems to produce more positive effects than sham acupuncture or no treatment for pain or quality of life.

Comparing CSRs of acupuncture in the past five years (2015–2019) with CSRs of acupuncture in the preceding years, we can find an increase of more than 10% in the coincidence rate of 9 of 16 items (1, 3, 4, 9, 10, 11, 12, 15, 16); among them, the items 10, 15 and 16 had an increase of more than 35%, and the items 4, 11 and 12 of more than 20%. Meanwhile, the coincidence rate of 3 items (5, 6, 14) has decreased, particularly item 6 with a decrease of more than 30%.

Since the large time span considered in this overview, the low quality rating of early published researches can be explained by the different methodological quality standards required in the preceding years. Improved standards in methodological evaluation can enhance the quality of CSRs and, in fact, the quality of acupuncture CSRs is increasing over time. On the basis of the quality evaluation performed in this article, we believe that some points are worthy of consideration and improvement, in particular:

explanation for the study design inclusion, reporting of the sources of funding, investigation and discussion of publication bias, reporting of potential sources of conflict of interest.

A more comprehensive search strategy including as much databases and languages as possible, especially Chinese, should be encouraged. International and interdisciplinary collaboration should be promoted when dealing with traditional medicines (e.g. Acupuncture) to integrate EBM methodology. Last but not least, the researchers should regularly update systematic reviews with the best methodological standards to improve quality overtime.

### Limitation

Since this study conducted a quality assessment of systematic reviews included in the Cochrane Library, it does not consider all the systematic reviews conducted on acupuncture. To avoid a misleading generalization, this limitation should be taken into consideration.

## Conclusions

In general, only half of the included CSRs were of medium to high quality according to the AMSTAR 2 evaluation tool, the main flaw being publication bias evaluation and discussion. Publication bias is very common in the Chinese literature and only in more recent years it has started to be considered, together with conflict of interest declarations and funding sources. Improvements in the evaluation of traditional Chinese medicine according to EBM methodology can promote the interdisciplinary collaboration and the quality of the studies.

## Supplementary information

**Additional file 1 Table S1**. Characteristics of the 50 included Cochrane Systematic Reviews [1–6]. **Table S2**. The interventions and controls table. **Table S3**. AMSTAR-2 scale (*) [table fully updated no track changes used].

## Data Availability

Please refer to the supporting Information.

## Abbreviations

CSR: Cochrane Systematic Review
EBM: Evidence-Based Medicine
TCM: Traditional Chinese Medicine
CDSR: Cochrane Database of Systematic Reviews
