# Supplementary material for: The quality of Cochrane systematic reviews of acupuncture: an overview
Source: BMC Complement Med Ther. 2020 Oct 14;20:307. doi: 10.1186/s12906-020-03099-9 (PMC7556594; doi:10.1186/s12906-020-03099-9)
Supplement: Supplementary file 1 — Additional file 1 Table S1. Characteristics of the 50 included Cochrane Systematic Reviews [1–6]. Table S2. The interventions and controls table. Table S3. AMSTAR-2 scale (*) [table fully updated no track changes used]. [file 12906_2020_3099_MOESM1_ESM.docx]

**Supporting Information**

**Table S1: Characteristics of the 50 included Cochrane Systematic Reviews** [1–6]

| **First Author - Year** | **First Publ. Year** | **Last Publ. Year** | **Last search date** | **Disease or Condition** | **Number of included trials** | **Number of included participants** | **Searched Databases** | **Chinese Databases** | **Methodological quality tool for the clinical trials assessment^(*)^** |
| --- | --- | --- | --- | --- | --- | --- | --- | --- | --- |
| Armour 2018 [7] | 2018 | - | 2017 | premenstrual syndrome | 5 | 277 | 11 | Yes | B |
| Casimiro 2005 [8] | 2002 | 2005 | 2005 | rheumatoid arthritis (RA) | 2 | 84 | 6 | No | M |
| Chen 2010 [9] | 2007 | 2010 | 2010 | Bell’s palsy | 6 | 537 | 6 | Yes | B |
| Cheng 2017 [10] | 2014 | 2017 | 2016 | acute hordeolum | 6 | 531 | 11 | Yes | B |
| Cheong 2013 [11] | 2008 | 2013 | 2013 | assisted reproductive technology (ART) | 20 | 4544 | 12 | No | B |
| Cheuk 2011 [12] | 2011 | - | 2011 | autism spectrum disorders (ASD) | 10 | 390 | 12 | Yes | B |
| Cheuk 2012 [13] | 2007 | 2012 | 2011 | insomnia | 33 | 2293 | 8 | Yes | B |
| Cheuk 2014 [14] | 2006 | 2014 | 2013 | epilepsy | 16 | 1486 | 8 | Yes | B |
| Choi 2018 [15] | 2018 | - | 2018 | carpal tunnel syndrome | 12 | 869 | 19 | Yes | B |
| Coyle 2012 [16] | 2002 | 2012 | 2012 | breech presentation | 8 | 1346 | 5 | No | B |
| Cui 2008 [17] | 2008 | - | 2008 | restless legs syndrome | 2 | 170 | 8 | Yes | J |
| Deare 2013 [18] | 2013 | - | 2012 | fibromyalgia | 9 | 395 | 8 | Yes | M |
| Dodin 2013 [19] | 2013 | - | 2013 | menopausal hot flushes | 16 | 1155 | 15 | Yes | B |
| Furlan 2005 [20] | 1999 | 2005 | 2003 | low-back pain | 35 | 2861 | 5 | No | M |
| Gates 2006 [21] | 2006 | - | 2005 | cocaine dependence | 7 | 1433 | 6 | No | M |
| Green 2002 [22] | 2002 | - | 2001 | lateral elbow pain | 4 | 239 | 4 | No | M |
| Green 2005 [23] | 1999 | 2005 | 2008 | shoulder pain | 9 | 525 | 4 | No | B |
| Ju 2017 [24] | 2017 | - | 2017 | neuropathic pain in adults | 6 | 462 | 9 | Yes | B |
| Kim 2014 [25] | 2014 | - | 2013 | acute ankle sprains in adults | 20 | 2012 | 10 | Yes | B |
| Kim 2016 [26] | 2016 | - | 2016 | chronic kidney disease (CKD) | 24 | 1787 | 6 | No | B |
| Kim 2018 [27] | 2018 | - | 2018 | symptomatic gastroparesis | 32 | 2601 | 14 | Yes | B |
| Lan 2014 [28] | 2014 | - | 2012 | functional dyspepsia | 7 | 542 | 8 | Yes | M |
| Law 2013[29] | 2007 | 2013 | 2013 | glaucoma | 1 | 33 | 15 | No | B |
| Lee 2015 [30] | 2004 | 2015 | 2014 | postoperative nausea and vomiting | 59 | 7667 | 6 | No | B |
| Lim 2016 [31] | 2011 | 2016 | 2015 | polycystic ovarian syndrome | 5 | 413 | 13 | Yes | B |
| Linde 2016 (EM) [32] | 2001 | 2016 | 2016 | prevention of episodic migraine (EM) | 22 | 4985 | 6 | No | B |
| Linde 2016 (TTH) [33] | 2009 | 2016 | 2016 | prevention of tension-type headache (TTH) | 12 | 2349 | 4 | No | B |
| Manheimer 2010 [34] | 2010 | - | 2008 | peripheral joint osteoarthritis | 16 | 3498 | 3 | No | B |
| Manheimer 2012 [35] | 2006 | 2012 | 2011 | irritable bowel syndrome | 17 | 1806 | 10 | Yes | B |
| Manheimer 2018 [36] | 2018 | 2018 | 2018 | hip osteoarthritis | 6 | 613 | 3 | No | B |
| McCarney 2003 [37] | 1998 | 2003 | 2008 | chronic asthma | 12 | 350 | 4 | No | J |
| Paley 2015 [38] | 2011 | 2015 | 2015 | cancer pain | 5 | 285 | 6 | No | B |
| Shen 2014 [39] | 2005 | 2014 | 2014 | schizophrenia | 30 | 2875 | 7 | No | B |
| Smith 2010 [40] | 2004 | 2010 | 2008 | depression | 30 | 2812 | 7 | Yes | B |
| Smith 2011 [41] | 2011 | - | 2011 | pain management in labour | 13 | 1986 | 4 | No | B |
| Smith 2013 [42] | 2000 | 2013 | 2012 | induction of labour | 14 | 2220 | 5 | No | B |
| Smith 2016 [43] | 2011 | 2016 | 2015 | dysmenorrhoea | 42 | 4640 | 8 | Yes | B |
| Smith 2017 [44] | 2001 | 2017 | 2016 | induction of labour | 22 | 3456 | 6 | No | B |
| Smith 2018 [45] | 2005 | 2017 | 2016 | depression | 64 | 7104 | 9 | No | B |
| Wang 2013 [46] | 2013 | - | 2013 | stress urinary incontinence in adults | 1 | 60 | 9 | Yes | B |
| Wei 2011 [47] | 2011 | - | 2011 | myopia in children and adolescents | 2 | 131 | 11 | Yes | B |
| White 2014 [48] | 2002 | 2014 | 2013 | smoking cessation | 38 | 6930 | 10 | Yes | B |
| Wong 2013 [49] | 2011 | 2013 | 2012 | acute management and rehabilitation of traumatic brain injury (TBI) | 4 | 294 | 22 | No | B |
| Xie 2008 [50] | 2008 | - | 2008 | dysphagia in acute stroke | 1 | 66 | 28 | Yes | B,J |
| Xu 2018 [51] | 2017 | 2018 | 2017 | acute stroke | 33 | 3946 | 9 | Yes | B |
| Yang 2016 [52] | 2006 | 2016 | 2016 | stroke rehabilitation | 31 | 2257 | 10 | Yes | B |
| Yang 2018 [53] | 2018 | - | 2017 | hypertension | 22 | 1744 | 8 | Yes | B |
| Zhang 2005 [54] | 2001 | 2005 | 2004 | acute stroke | 14 | 1208 | 8 | Yes | M |
| Zhang 2018 [55] | 2018 | - | 2018 | cancer patients after radiotherapy or chemotherapy | 29 | 2569 | 10 | Yes | M |
| Zhu 2011 [56] | 2011 | - | 2010 | pain in endometriosis | 1 | 67 | 8 | No | B |
| (*) B: Bias risk assessment tools of Cochrane Handbook of Systematic Reviewers; J: Jadad Score; M: The methodological quality tool for the clinical trials assessment was unclear and incomplete. | | | | | | | | | |

**Table S2: The interventions and controls table**

| **Author - Year** | **Number of interventions** | **Interventions** | **Control measures^(*)^** |
| --- | --- | --- | --- |
| Armour 2018 [7] | 9 | 1. body acupuncture  2. electro-acupuncture  3. scalp acupuncture  4. ear acupuncture  5. acupressure  6. electromagnetic acupuncture  7. pyonex  8. intradermal needling  9. moxibustion | 1,5,6,7 |
| Casimiro 2005 [8] | 1 | acupuncture applications using any combination of parameters | 2 |
| Chen 2010 [9] | 1 | all types of acupuncture treatments | 1,2,5 |
| Cheng 2017 [10] | 2 | 1. bloodletting methods at the ear apex  2. needling acupuncture at other body points such as points at upper back or at upper arm | 1,5,6 |
| Cheong 2013 [11] | 3 | 1. acupuncture + Assisted Reproductive Technology (ART) vs no treatment, placebo, sham acupuncture + ART  2. acupuncture alone vs no treatment, placebo, sham acupuncture + ART  3. acupuncture vs acupuncture + ART | 1,2,5,6 |
| Cheuk 2011 [12] | 3 | 1. acupressure  2. laser acupuncture  3. electroacupuncture | 1,2,3,6 |
| Cheuk 2012 [13] | 6 | 1. acupressure  2. laser acupuncture  3. electroacupuncture  4. auricular therapy  5. magnetic acupressure  6. transcutaneous electrical acupoints stimulation | 1,2,3,5,6 |
| Cheuk 2014 [14] | 4 | 1. acupressure  2. laser acupuncture  3. electroacupuncture  4. catgut implantation | 1,2,3,5,6,7 |
| Choi 2018 [15] | 1 | 1. all types of acupuncture  2. acupressure  3. laser acupuncture  4. moxibustion | 1,2,3,5,7 |
| Coyle 2012 [16] | 2 | 1. moxibustion (entailing application of heat to the acupoint Zhiyin BL67) alone  2. moxibustion in combination with acupuncture or postural techniques | 1,4,5 |
| Cui 2008 [17] | 8 | 1. body acupuncture  2. auricular acupuncture  3. scalp acupuncture  4. electroacupuncture  5. laser acupuncture  6. dermal needle therapy  7. acupoint injection therapy  8. acupressure therapy | 1,2,3,6 |
| Deare 2013 [18] | 1 | acupuncture that breaks the skin for therapeutic benefit | 1,2,3,4,5,6 |
| Dodin 2013 [19] | 7 | 1. TCM acupuncture  2. electroacupuncture  3. acupressure  4. laser acupuncture  5. ear acupuncture  6. moxibustion  7. scalp acupuncture | 1,2,5,6 |
| Furlan 2005 [20] | 1 | all categories of acupuncture | 1,2,5,6 |
| Gates 2006 [21] | 1 | auricular acupuncture using any method | 1,6 |
| Green 2002 [22] | 2 | 1. acupuncture vs placebo  2. acupuncture vs another modality | 2,5 |
| Green 2005 [23] | 1 | all categories of acupuncture | 1,2,4,5 |
| Ju 2017 [24] | 1 | 1. any stimulation based on needle insertion  2. electro-acupuncture  3. warm needling | 3,5,6 |
| Kim 2014 [25] | 6 | 1. needle acupuncture  2. electroacupuncture  3. laser acupuncture  4. pharmaco-acupuncture  5. non-penetrating acupuncture point stimulation (e.g. acupressure and magnets)  6. moxibustion | 1,2,3,5 |
| Kim 2016 [26] | 4 | 1. electro-stimulation using a penetrating needle or non-penetrating pad, laser, manual acupressure or acupressure with other devices  2. moxibustion (heating the herb Mugwort)  3. other types of heat stimulation on acupoints  4. studies investigating combinations of acupuncture interventions | 5,6,7 |
| Kim 2018 [27] | 1 | the penetration of skin or muscle by needles, regardless of the stimulation method | 1,5,6 |
| Lan 2014 [28] | 2 | 1. manual acupuncture  2. electroacupuncture | 1,2,3,6 |
| Law 2013[29] | 3 | 1. needle insertion  2. acupressure  3. surface electrical and laser stimulation | 1,2,4,5 |
| Lee 2015 [30] | 8 | 1. acupuncture  2. electroacupuncture  3. laser acupuncture  4. transcutaneous electrical stimulation  5. conventional peripheral nerve stimulation  6. acu-stimulation device  7. acupressure  8. capsicum plaster | 3,6 |
| Lim 2016 [31] | 2 | 1. body needling  2. electroacupuncture | 1,2,3,5,6 |
| Linde 2016 (EM) [32] | 1 | acupuncture | 1,3,6 |
| Linde 2016 (TTH) [33] | 1 | acupuncture | 1,5,6 |
| Manheimer 2010 [34] | 1 | traditional acupuncture | 3,5,6 |
| Manheimer 2012 [35] | 2 | 1. Traditional Chinese Medicine (TCM) acupuncture  2. moxibustion | 1,2,3,5,6 |
| Manheimer 2018 [36] | 1 | traditional Chinese medicine needle acupuncture | 1,5,6,7 |
| McCarney 2003 [37] | 3 | 1. needle acupuncture  2. lasers stimulation  3. formula acupuncture | 6,7 |
| Paley 2015 [38] | 3 | 1. manual acupuncture  2. auricular acupuncture  3. electroacupuncture | 1,5,6,7 |
| Shen 2014 [39] | 1 | all categories of acupuncture | 1,2,3,5 |
| Smith 2010 [40] | 3 | 1. manual acupuncture  2. electroacupuncture  3. laser acupuncture | 1,2,3,5,6,7 |
| Smith 2011 [41] | 1 | any type of acupuncture or acupressure | 1,2,5 |
| Smith 2013 [42] | 3 | 1. manual acupuncture  2. electroacupuncture  3. manual and electroacupuncture | 1,2,5,6 |
| Smith 2016 [43] | 3 | 1. manual acupressure  2. electroacupuncture  3. moxa (warming needle) | 1,2,3,5 |
| Smith 2017 [44] | 4 | 1. manual acupuncture  2. laser acupuncture  3. electro-acupuncture  4. acupressure | 1,2,5,6,7 |
| Smith 2018 [45] | 3 | 1. manual acupuncture  2. electro-acupuncture  3. laser acupuncture | 1,2,3,4,5,6 |
| Wang 2013 [46] | 7 | 1. scalp acupuncture  2. body acupuncture  3. electroacupuncture  4. warm acupuncture  5. elongated needle  6. auricular acupuncture  7. fire needle | 1,2,5 |
| Wei 2011 [47] | 6 | 1. acupressure  2. auricular acupuncture  3. conventional acupuncture (needle insertion)  4. electroacupuncture  5. laser acupuncture  6. eye exercise | 1,2,5,6 |
| White 2014 [48] | 6 | 1. facial acupuncture  2. auricular acupuncture alone  3. some form of continuous stimulation  4. combined body and auricular acupuncture  5. continuous stimulation with either indwelling needles or seeds  6. facial, body, indwelling, and sham auricular acupuncture in different groups | 1,5,6,7 |
| Wong 2013 [49] | 6 | 1. scalp acupuncture  2. body acupuncture  3. auricular acupuncture  4. tongue acupuncture  5. injection acupuncture  6. electro acupuncture | 5 |
| Xie 2008 [50] | 2 | 1. traditional acupuncture  2. contemporary acupuncture | 1,2,6,7 |
| Xu 2018 [51] | 2 | 1. manual acupuncture  2. electro-acupuncture | 1,2,3,5,6,7 |
| Yang 2016 [52] | 2 | 1. traditional acupuncture  2. contemporary acupuncture | 2,3,5,6 |
| Yang 2018 [53] | 1 | needle insertion at acupuncture points, pain points, or trigger points | 1,3,6 |
| Zhang 2005 [54] | 2 | 1. traditional acupuncture  2. contemporary acupuncture | 1,2,6 |
| Zhang 2018 [55] | 1 | any type of moxibustion treatment | 1,5 |
| Zhu 2011 [56] | 3 | 1. body acupuncture with needle insertion at traditional acupoints or insertion at non-traditional points, also called ‘Ashi’ or tender areas  2. scalp or auricular acupuncture  3. electroacupuncture | 1,2,5,6 |
| (*) 1. blank (none intervention); 2. Placebo; 3. Drugs; 4. needles (different styles of acupuncture); 5. conventional/other treatment; 6. sham acupuncture; 7. sham stimulation (sham laser, sham moxibustion, sham electro-stimulation, sham acupressure, other) | | | |

**Table S3: AMSTAR-2 scale (*) [table fully updated no track changes used]**

| First Author - Year | 1. Did the research questions and inclusion criteria for the review include the components of PICO? | **2. Did the report of the review contain an explicit statement that the review methods were established prior to the conduct of the review and did the report justify any significant deviations from the protocol?** | 3. Did the review authors explain their selection of the study designs for inclusion in the review? | **4. Did the review authors use a comprehensive literature search strategy?** | 5. Did the review authors perform study selection in duplicate? | 6. Did the review authors perform data extraction in duplicate? | **7. Did the review authors provide a list of excluded studies and justify the exclusions?** | 8. Did the review authors describe the included studies in adequate detail? | **9. Did the review authors use a satisfactory technique for assessing the risk of bias (RoB) in individual studies that were included in the review?** | 10. Did the review authors report on the sources of funding for the studies included in the review? | **11. If meta-analysis was performed did the review authors use appropriate methods for statistical combination of results?** | 12. If meta-analysis was performed, did the review authors assess the potential impact of RoB in individual studies on the results of the meta-analysis or other evidence synthesis? | **13. Did the review authors account for RoB in individual studies when interpreting/discussing the results of the review?** | 14. Did the review authors provide a satisfactory explanation for, and discussion of, any heterogeneity observed in the results of the review? | **15. If they performed quantitative synthesis did the review authors carry out an adequate investigation of publication bias (small study bias) and discuss its likely impact on the results of the review?** | 16. Did the review authors report any potential sources of conflict of interest, including any funding they received for conducting the review? | **RATING OVERALL CONFIDENCE** |
| --- | --- | --- | --- | --- | --- | --- | --- | --- | --- | --- | --- | --- | --- | --- | --- | --- | --- |
| Armour 2018 [7] | Y | **Y** | No | **Y** | Y | Y | **Y** | Y | **Y** | Y | **Y** | Y | **Y** | Y | **Y** | Y | **H** |
| Casimiro 2005 [8] | No | **Y** | No | **PY** | Y | Y | **Y** | Y | **Y** | No | **Y** | Y | **Y** | Y | **No** | No | **L** |
| Chen 2010 [9] | Y | **Y** | No | **PY** | Y | Y | **Y** | Y | **Y** | No | **N-MA** | N-MA | **Y** | Y | **N-MA** | No | **M** |
| Cheng 2017 [10] | Y | **Y** | No | **PY** | Y | Y | **Y** | Y | **Y** | Y | **Y** | Y | **Y** | Y | **Y** | Y | **H** |
| Cheong 2013 [11] | Y | **Y** | No | **Y** | Y | Y | **Y** | Y | **Y** | Y | **Y** | Y | **Y** | Y | **No** | No | **L** |
| Cheuk 2011 [12] | Y | **Y** | No | **Y** | Y | Y | **Y** | Y | **Y** | No | **Y** | Y | **Y** | Y | **No** | No | **L** |
| Cheuk 2012 [13] | Y | **Y** | No | **PY** | Y | Y | **Y** | Y | **Y** | No | **Y** | Y | **Y** | Y | **Y** | No | **M** |
| Cheuk 2014 [14] | Y | **Y** | No | **Y** | Y | Y | **Y** | Y | **Y** | No | **Y** | Y | **Y** | Y | **No** | No | **L** |
| Choi 2018 [15] | Y | **Y** | No | **Y** | Y | Y | **Y** | Y | **Y** | Y | **Y** | Y | **Y** | Y | **Y** | Y | **H** |
| Coyle 2012 [16] | Y | **Y** | No | **Y** | Y | Y | **Y** | Y | **Y** | No | **Y** | Y | **Y** | Y | **No** | No | **L** |
| Cui 2008 [17] | Y | **Y** | No | **PY** | Y | Y | **Y** | Y | **PY** | No | **Y** | Y | **Y** | Y | **No** | No | **L** |
| Deare 2013 [18] | Y | **Y** | No | **PY** | Y | Y | **Y** | Y | **Y** | Y | **Y** | Y | **Y** | Y | **Y** | No | **M** |
| Dodin 2013 [19] | Y | **Y** | No | **PY** | Y | Y | **Y** | Y | **Y** | Y | **Y** | Y | **Y** | Y | **No** | No | **L** |
| Furlan 2005 [20] | No | **Y** | No | **No** | Y | Y | **Y** | Y | **Y** | Y | **Y** | Y | **Y** | Y | **No** | Y | **CL** |
| Gates 2006 [21] | Y | **Y** | No | **PY** | Y | Y | **Y** | Y | **Y** | No | **Y** | Y | **Y** | Y | **No** | No | **L** |
| Green 2002 [22] | Y | **Y** | No | **No** | Y | Y | **Y** | Y | **PY** | No | **N-MA** | N-MA | **Y** | Y | **N-MA** | No | **L** |
| Green 2005 [23] | Y | **Y** | No | **PY** | Y | Y | **Y** | Y | **PY** | No | **Y** | Y | **Y** | Y | **No** | No | **L** |
| Ju 2017 [24] | Y | **Y** | No | **PY** | Y | Y | **Y** | Y | **Y** | Y | **Y** | Y | **Y** | Y | **Y** | Y | **H** |
| Kim 2014 [25] | Y | **Y** | No | **PY** | Y | Y | **Y** | Y | **Y** | No | **Y** | Y | **Y** | Y | **No** | Y | **L** |
| Kim 2016 [26] | Y | **Y** | No | **PY** | Y | Y | **Y** | Y | **Y** | Y | **Y** | Y | **Y** | Y | **No** | No | **L** |
| Kim 2018 [27] | Y | **Y** | Y | **Y** | Y | No | **Y** | Y | **Y** | Y | **Y** | Y | **Y** | Y | **Y** | Y | **H** |
| Lan 2014 [28] | Y | **Y** | No | **PY** | Y | Y | **Y** | Y | **Y** | No | **Y** | Y | **Y** | Y | **No** | No | **L** |
| Law 2013[29] | Y | **Y** | No | **PY** | Y | Y | **Y** | Y | **Y** | Y | **N-MA** | N-MA | **Y** | Y | **N-MA** | Y | **H** |
| Lee 2015 [30] | Y | **Y** | No | **PY** | Y | Y | **Y** | Y | **Y** | Y | **Y** | Y | **Y** | Y | **Y** | Y | **H** |
| Lim 2016 [31] | Y | **Y** | No | **PY** | No | No | **Y** | Y | **Y** | No | **Y** | Y | **Y** | Y | **No** | No | **L** |
| Linde 2016 (EM) [32] | Y | **Y** | No | **Y** | Y | Y | **Y** | Y | **Y** | Y | **Y** | Y | **Y** | Y | **No** | Y | **L** |
| Linde 2016 (TTH) [33] | Y | **Y** | No | **Y** | Y | Y | **Y** | Y | **Y** | No | **Y** | Y | **Y** | Y | **Y** | Y | **M** |
| Manheimer 2010 [34] | Y | **Y** | No | **No** | Y | Y | **Y** | Y | **Y** | Y | **Y** | Y | **Y** | Y | **Y** | No | **L** |
| Manheimer 2012 [35] | Y | **Y** | No | **PY** | Y | Y | **Y** | Y | **Y** | Y | **Y** | Y | **Y** | Y | **No** | Y | **L** |
| Manheimer 2018 [36] | Y | **Y** | Y | **PY** | Y | Y | **Y** | Y | **Y** | Y | **Y** | Y | **Y** | Y | **No** | Y | **L** |
| McCarney 2003 [37] | No | **Y** | No | **No** | Y | Y | **Y** | Y | **PY** | No | **Y** | Y | **Y** | Y | **No** | Y | **CL** |
| Paley 2015 [38] | Y | **Y** | No | **PY** | Y | Y | **Y** | Y | **Y** | No | **N-MA** | N-MA | **Y** | Y | **N-MA** | Y | **M** |
| Shen 2014 [39] | Y | **Y** | No | **PY** | Y | Y | **Y** | Y | **Y** | No | **Y** | Y | **Y** | Y | **Y** | No | **M** |
| Smith 2010 [40] | Y | **Y** | No | **PY** | Y | Y | **Y** | Y | **Y** | No | **Y** | Y | **Y** | Y | **No** | No | **L** |
| Smith 2011 [41] | Y | **Y** | No | **PY** | Y | Y | **Y** | Y | **Y** | No | **Y** | Y | **Y** | Y | **No** | No | **L** |
| Smith 2013 [42] | Y | **Y** | No | **PY** | Y | Y | **Y** | Y | **Y** | No | **Y** | Y | **Y** | Y | **No** | Y | **L** |
| Smith 2016 [43] | Y | **Y** | No | **PY** | Y | Y | **Y** | Y | **Y** | Y | **Y** | Y | **Y** | Y | **No** | No | **L** |
| Smith 2017 [44] | Y | **Y** | No | **PY** | Y | No | **Y** | Y | **Y** | Y | **Y** | Y | **Y** | No | **Y** | No | **M** |
| Smith 2018 [45] | Y | **Y** | Y | **Y** | Y | No | **Y** | Y | **Y** | No | **Y** | Y | **Y** | Y | **Y** | No | **H** |
| Wang 2013 [46] | Y | **Y** | No | **PY** | Y | Y | **Y** | Y | **Y** | No | **N-MA** | N-MA | **Y** | Y | **N-MA** | Y | **M** |
| Wei 2011 [47] | Y | **Y** | No | **PY** | Y | Y | **Y** | Y | **Y** | No | **N-MA** | N-MA | **Y** | Y | **N-MA** | Y | **M** |
| White 2014 [48] | Y | **Y** | No | **PY** | Y | Y | **Y** | Y | **Y** | No | **Y** | Y | **Y** | Y | **Y** | Y | **M** |
| Wong 2013 [49] | No | **Y** | No | **PY** | Y | Y | **Y** | Y | **Y** | No | **N-MA** | N-MA | **Y** | Y | **N-MA** | Y | **M** |
| Xie 2008 [50] | Y | **Y** | No | **Y** | Y | Y | **Y** | Y | **PY** | No | **N-MA** | N-MA | **Y** | Y | **N-MA** | No | **M** |
| Xu 2018 [51] | Y | **Y** | No | **PY** | Y | No | **Y** | Y | **Y** | No | **Y** | Y | **Y** | Y | **Y** | Y | **M** |
| Yang 2016 [52] | Y | **Y** | No | **Y** | Y | Y | **Y** | Y | **Y** | No | **Y** | Y | **Y** | Y | **Y** | No | **M** |
| Yang 2018 [53] | Y | **Y** | No | **PY** | Y | No | **Y** | Y | **Y** | Y | **Y** | Y | **Y** | Y | **Y** | Y | **M** |
| Zhang 2005 [54] | Y | **Y** | No | **PY** | Y | Y | **Y** | Y | **Y** | No | **Y** | Y | **Y** | Y | **No** | No | **L** |
| Zhang 2018 [55] | Y | **Y** | No | **Y** | Y | Y | **Y** | Y | **Y** | No | **Y** | Y | **Y** | No | **No** | Y | **L** |
| Zhu 2011 [56] | Y | **Y** | No | **PY** | Y | Y | **Y** | Y | **Y** | No | **N-MA** | N-MA | **Y** | Y | **N-MA** | No | **M** |
| **% by domain** | 92% | **100%** | 6% | **92%** | 98% | 88% | **100%** | 100% | **90%** | 38% | **100%** | 100% | **100%** | 96% | **52%** | 48% |  |

**(*) Critical Domains in Bold; Y: Yes, N: No, PY: Partial Yes, N-MA: No Meta-Analysis Conducted.**
